# 高分辨分离分析新技术在食品安全检测领域的应用进展

**DOI:** 10.3724/SP.J.1123.2023.08026

**Published:** 2023-11-08

**Authors:** Yan SUN, Yun XIE, Xiuli XU, Mingqian TAN, Feng ZHANG

**Affiliations:** 1.中国检验检疫科学研究院, 北京 100176; 1. Chinese Academy of Inspection and Quarantine, Beijing 100176, China; 2.大连工业大学食品学院, 辽宁 大连 116034; 2. School of Food Science and Technology, Dalian Polytechnic University, Dalian 116034, China

**Keywords:** 在线质谱耦合技术, 高分辨筛查技术, 微型质谱仪, 食品安全, on-line mass spectrometry coupling technology, high resolution screening technology, micro mass spectrometer, food safety

## Abstract

近十年来,质谱技术在食品检验学中得到了广泛应用,同时仪器的不断改进是分析化学技术取得突破的关键。本文讨论了高分辨分离分析技术在食品安全检测领域的最新进展与应用(2013-2023年);重点介绍了在线质谱耦合技术、高分辨筛查技术以及微型质谱仪等创新发展技术;最后对食品安全检测新装置的开发进行了展望。

## Abstract (en)

In the last decade, mass spectrometry (MS) has been widely used in food inspection science, and the continuous improvement of instruments is the key to the breakthrough of analytical chemistry technology. This paper discusses the latest progress and application of high resolution separation and analysis technology in food safety field (2013-2023), focusing on the innovative development technologies such as on-line MS coupling technology, high resolution screening technology and micro mass spectrometer. Finally, the development of new device in the field of food safety detection is prospected.

食品安全与质量对全球经济、人类健康和国土安全至关重要。然而,由于食品种类的多样性及化学成分的复杂性,微生物病原体、重金属、食品添加剂、生物毒素、农/兽药,甚至食品包装材料微塑料成分等多种痕量污染物的快速鉴别成为现代社会食品安全分析的一大挑战^[1]^。除了食品化学污染外,食品还面临着非法掺假、降解变质等,虽然传统技术(如色谱分析法、光谱分析法等)可以实现食品中目标化合物的检测,但繁琐的样品前处理过程(分离、提取、净化、富集等)已不适用于当代食品检测学中对风险物质的快速高通量筛查^[2]^。

因此,针对复杂化学混合物中分子离子的筛选,离子迁移谱(ion mobility spectrometry, IMS)作为一种快速分离技术,新增了一维离子淌度信息——碰撞横截面积,其测量与气态离子的大小、形状和所带电荷有关,不受样品基质影响,检测信噪比也有所提高,因此能够有效分辨同分异构体、多电荷态物质等^[3]^。同时高分辨MS作为分析复杂样品的常用设备,具有在原子和分子水平上进行多组分分析的优点,且各种类型的离子碰撞解离技术极大地扩展了MS在食品分析方面的应用^[4]^。一方面,质谱数据库的构建以及机器学习算法程序的应用,大大提高了食品中未知风险成分的高分辨筛查与预测能力;另一方面,敞开式离子化质谱法(ambient mass spectrometry, AMS)作为传统MS的一个重要的创新突破,是一种快速有效的复杂样品直接分析方法,因此成为高通量定性分析、无损反应监测的绝佳选择^[5]^。

高分辨MS作为实验室仪器在分析应用领域有着较大发展,但也存在体积庞大、价格昂贵、操作复杂、不能随时移动等局限性,因此无法在食品环境污染、食品风险因子、突发应急监测等需要进行现场快速检测的领域得到有效应用。目前质谱仪器正向高效率、便携化、可视化方面发展,出现了微型质谱仪。未来开发无需样品前处理、可由非专业人员操作、具备高分辨分离分析性能的微型质谱仪,对满足原位、实时、无损的食品现场快检十分重要^[6]^。

本文重点概述了近十年高分辨分离分析技术在食品安全领域的最新进展与应用,分别通过在线质谱耦合技术、高分辨筛查技术以及微型质谱仪3大领域展开介绍,并对食品安全检测新装置的前景进行了展望。

## 1 在线质谱耦合技术

MS是在线过程优化和智能控制的基本仪器,在线质谱法的优势是能够表征化学反应过程,如化学产物和杂质的形成以及底物的消耗,在线质谱技术作为一种高灵敏检测技术,已由推测化学反应机理研究逐渐向痕量物质的实时快速检测和准确定量方面应用^[7]^。为了实现各种设备与质谱的在线联用,最关键的问题是在两个设备之间开发合适的接口,以解决大气压气流对质谱检测器造成的真空冲击。目前MS已实现与色谱分离技术(例如超高效液相色谱、气相色谱、毛细管电泳、超临界流体色谱)串联,但涉及富集提取-色谱分离-质谱检测的耗时过程^[8]^。然而,随着IMS与AMS的出现与发展,在线质谱法有了新的可供选择的耦合方式,并已有成功应用于小型化设备现场分析的案例^[9]^。

### 1.1 离子迁移谱法

IMS的分离原理是根据气相离子的质量、形状、大小和电荷在电场中的迁移率不同来分离的。因此,结构相似但具有不同电荷的离子可以通过IMS进行分离。然而IMS仍然是一种低分辨率技术,因此,越来越多的研究人员选择将IMS与高分辨MS串联使用,IMS-MS联用技术最初在2000年左右出现,但不同类型的IMS-MS测量离子迁移率的能力不同,大致分为漂移管离子迁移谱法(drift tube IMS, DTIMS)、吸入离子迁移谱法(aspiration IMS, AIMS)、行波离子迁移谱法(traveling wave IMS, TWIMS)、场不对称形离子迁移谱法(field asymmetric IMS, FAIMS)和捕获离子迁移谱法(trapped IMS, TIMS)。

#### 1.1.1 漂移管离子迁移谱法

DTIMS使用恒定电场来分离离子,缺点是灵敏度低,因此需要与MS串联使用。IMS仪器的工作时间为毫秒,而MS的工作时间为秒,因此通常使用快速扫描质谱仪,如飞行时间质谱仪(time of flight, TOF)作为耦合的高分辨MS。Gloess等^[10]^采用电晕放电电离-DTIMS-TOF联用技术,在正离子和负离子模式下监测咖啡烘焙过程中形成的150多种挥发性有机化合物。结果表明,该在线监测模式检测到一些重要的香气活性物质,如烷基吡嗪异构体,其随着焙烧过程的不同时间段表现出不同的强度特征。由此可知,DTIMS-TOF在线耦合技术为这类含异构体的香气活性化合物的复杂化学分析提供了一个简单快捷的高分辨率监测策略,且DTIMS实验中产生的迁移率趋势有利于识别多种同分异构体。

#### 1.1.2 吸入离子迁移谱法

AIMS目前已成功从实验室转型为现场分析测试设备。Potter等^[11]^使用了气相色谱-大气压化学电离-AIMS-TOF MS耦合系统,对一系列市售食用油中的脂肪酸进行特异性分离鉴别,并开发了一种全面指纹识别技术。这种技术与传统使用火焰电离检测或单四极杆质谱检测脂肪酸相比,无需衍生为脂肪酸甲酯来检测,从而提高了脂肪酸鉴定的特异性和定量的可信度。由此可见,AIMS-MS串联大大提高了化合物的选择性,在研究食品来源、食品掺假等方面具有巨大的潜力。

#### 1.1.3 场不对称形离子迁移谱法

FAIMS又称差分迁移谱法,是利用离子迁移率从低电场到高电场的变化来分离离子,克服了离子迁移率相似的物质无法分离的缺点。然而,FAIMS技术的分辨率较低,需依赖FAIMS-MS串联技术提高分辨率。同时随着FAIMS接口的小型化,与质谱成像的耦合可以提高特定分子类别的检测与成像。

Feider等^[12]^报道了解吸电喷雾电离和液体微临界表面取样探针与FAIMS装置的耦合技术,可增强生物组织样品中的单电荷以及多电荷代谢物的成像。优化后的FAIMS参数提高了代谢物空间分布的可视化,最重要的是可以监测到单使用质谱成像无法检测到的成分种类。由此可知,FAIMS-质谱成像耦合技术可实现食品中多电荷脂质和蛋白质成分的检测,且FAIMS和MS仪器的无缝集成极大地拓展了FAIMS在食品组学方面的应用和前景。

#### 1.1.4 行波离子迁移谱法

TWIMS技术推动了无损离子结构的检测技术的发展,同时TWIMS与TOF质谱仪器联用,对序列输出离子的信噪比有显著的提升。但单级TWIMS产生周期性选择离子的连续输出,也产生非选择离子的脉冲输出,导致背景信号增强。理论上,两个TWIMS的正交联用可以消除这种非选择离子脉冲的输出。

Vidal-de-Miguel等^[13]^介绍了一种两级TWIMS与大气压界面质谱耦合技术,该技术可以连续输出选定迁移率的离子。结果表明,采用了两级TWIMS的过滤方式,可大大减少单级TWIMS分离产生的背景干扰,且IMS-IMS预过滤方法可作为提高样品分离能力的一种非常有效的方法。

#### 1.1.5 捕获离子迁移谱法

与其他IMS技术相比,TIMS除了分辨分离能力高和测量离子碰撞横截面积准确度高的优点外,还具有在复杂样品中靶向分析分子离子的优势,因此TIMS-MS多用于快速气相色谱分离和分子结构解析。Djambazova等^[14]^利用基质辅助激光解吸电离质谱成像耦合TIMS技术对脂质异构体(手性异构体、酰基链异构体、双键位置和立体异构体)进行分离和鉴定。结果表明,质谱成像-TIMS耦合技术可以直接对脂质异构体进行原位分离和空间分布可视化成像。由此可知,对于大分子、蛋白质及其复合物的异构化,TIMS-MS有表征化合物结构多样性的潜力。

### 1.2 敞开式离子化质谱法

AMS是一种无需样品预处理,直接进行表面采样,几秒钟内即可进行成分研究的实时解吸电离分析技术,可以与大多数带有大气压接口的高分辨率MS耦合并进行精确的质量分析,极大地扩展了食品现场快检应用领域。最早出现的AMS技术分别是2004年和2005年提出的解吸电喷雾电离和实时直接分析电离技术,之后陆续出现了许多不同的AMS技术,目前已有40多种类型。根据电离能源(如电、光、热、声等)的能量形式差异,AMS大致分为喷雾电离、电场电离(电晕/辉光放电、等离子体等)、光电离和热电离^[15]^。

#### 1.2.1 喷雾电离

喷雾电离的机制是将样品本身(切割后产生尖锐的尖端)作为固体底物,在样品上施加高压和一些溶剂,分析物溶解到溶剂中,接着液相分析物分子在高电场下直接电离,喷射并产生带电液滴和离子,随后进入MS分析。由于实际样品以固体、液体、气体、胶体甚至非均相形式存在,为了适应不同的样品分析,以喷雾电离为基础的各种AMS技术相继出现,如解吸电喷雾电离、萃取电喷雾电离、解吸声波喷雾电离、气流辅助电离、熔滴电喷雾电离、探针电喷雾电离、组织喷雾电离等。Cai等^[16]^采用纳米萃取电喷雾电离与MS耦合技术对啤酒样品中的组胺进行实时、快速检测,无需繁琐的样品前处理操作。由此可见,喷雾电离的主要优点是能够对样品表面进行电离,实现快速定性分析,并利用软电离来限制目标分析物的破碎程度;主要局限是当使用不合适的溶剂系统时,分析物的提取会减少,导致灵敏度降低。

#### 1.2.2 电场电离

电场电离的机制是利用大气压化学电离和大气压电晕放电与辉光放电产生的等离子体进行电离,常见技术如低温等离子体电离、介质阻挡放电电离和实时直接分析电离等。实时直接分析电离属于辉光放电产生等离子体电离,原理是通过对气体He或N_2_分子进行放电形成等离子体激发态,这些物质被加热的气流带到样品中,从样品中解吸和电离分析物,随后进入MS分析。实时直接分析电离作为一种气相电离技术,很少观察到由液相电离技术形成的复杂簇离子、金属加合物离子和多电荷离子等,但相对缺点是放电气体对解吸分析物的稀释以及高温下不可避免的离子破碎,因此更适用于小分子化合物的快速电离。Crawford等^[17]^采用实时直接分析电离与MS串联的方法,对食品包装中邻苯二甲酸盐进行快速定性分析。结果表明,该方法成功应用于监测各种食品包装以及运动器材中邻苯二甲酸盐的存在,但其定量能力仍相对不足。

#### 1.2.3 光电离

光电离分为直接光电离(以紫外、红外和激光为能量源)和基质辅助激光电离,通常不需要高电压、喷雾溶剂或气体来帮助电离,并且功耗低。该技术可使样品快速气化和电离,常见光电离技术包括解吸常压光电离、常压激光基质辅助解吸电离、常压激光解吸电离等。Aiello等^[18]^报道了一种快速、灵敏的激光基质辅助解吸电离与MS串联的方法,并对藏红花的真伪进行了定性鉴别。研究选择藏红花素作为藏红花真伪识别生物标志物,并用姜黄素作掺假物对市售藏红花的掺假率进行了评价。结果表明,该方法是表征食品中真伪生物标志物的一种强有力的方法,具有很高的化学敏感性,可作为食品真伪识别工具。

#### 1.2.4 热电离

热电离技术利用高温和热辐射光子的能量来诱导分析物电离。如热解吸化学电离是通过可离子化试剂热解产生高浓度试剂离子,然后与样品中的待测分子发生碰撞进行电离。该技术无需高压气体,且灵敏度高。其他常见的热电离技术包括激光二极管热解吸、常压热解吸电离、快速蒸发电离等。Balog等^[19]^提出了一种快速蒸发电离质谱法,能够在小于5 s的时间内记录肉类标本的质谱剖面。结果表明,该方法对来自不同物种(马、牛和鹿肉)肉类的混合肉饼鉴别具有较高的准确性,且可以通过提供原位、实时的分子解析信息对肉制品进行快速有效的质量判定,检测过程绿色无污染。

## 2 高分辨筛查技术

高分辨筛查技术一般分为靶向筛查和非靶向筛查,靶向筛查可减少一定干扰离子的存在,但不适合在复杂食品样品中发现潜在风险化合物;非靶向筛查可获得样本所有离子的碎片信息,更适合复杂样本的高通量筛查分析。但由于样品制备、有机溶剂、采集方法和数据分析等差异,不同高分辨MS用于筛查风险物质的方法准确性存在很大差异。因此,构建高质量质谱数据库,尽可能去除不同仪器与实验操作的干扰,减少对参考标准品的依赖,对未知化合物的有效筛查至关重要。

### 2.1 质谱数据库

一直以来,在质谱的特征二级碎片数据库中进行食品未知成分碎片的相似性搜索很有意义。随着质谱数据库中化合物数量与种类的不断扩大,从文献中获得的二级质谱信息对于建立一个庞大的高分辨质谱数据库非常有价值。Li等^[20]^使用全谱采集的气相色谱-四极杆-TOF质谱体系,建立了439种农药的TOF精确质量数据库和图谱库。结果表明,该质谱体系可实现果蔬中439种农药的快速分析,成为监测水果和蔬菜中农药残留的可靠分析方法。Li等^[21]^采用超高效液相色谱-三重四极杆/线性离子阱质谱技术,建立了猪肉中磺胺类、喹诺酮类、*β*-受体激动剂、硝基咪唑等204种多残留兽药鉴定的方法。结果表明,该方法建立的204种兽药二级质谱碎片数据库可加强对肉类食品中可疑风险物的定性筛查。但来源于文献的信息包含了各种类型的MS仪器,不同的离子源、前体加合物和碰撞能的影响已远远超出质谱碎片数据库匹配的范围,因此仅以保留时间以及特征二级质谱数据库进行匹配筛查会出现很多假阳性峰值。

为了进一步提供结构解析的准确性,除了光谱特征和二级质谱的特征质荷比片段,中性丢失、母离子以及特征质荷比之间差异的信息也作为分子结构解析的考察点。因此研究不同种类化学物质在不同离子源作用下的裂解规律非常重要。Guo等^[22]^采用敞开式电离-四极杆-轨道阱质谱法(AI-Q-Orbitrap),根据酸性化合物的特征子离子和中性丢失的结构信息,对不同酸性化合物的结构相似性和多样性进行解析。该研究根据酸性化合物在二级质谱高碰撞解离过程中的行为,提出了3种裂解规律:(1)电荷驱动下的裂解和CO_2_的丢失;(2)六元环重排和C*α*-C*β*裂解;(3)消除重排和H_2_O、CO_2_和CO的连续丢失,裂解途径详见图1。结果表明,AI-Q-Orbitrap方法可用于酒和茶中的单酸、双酸、多酸和酚酸等多类酸性化合物的高通量筛查与鉴定。

**图1:**
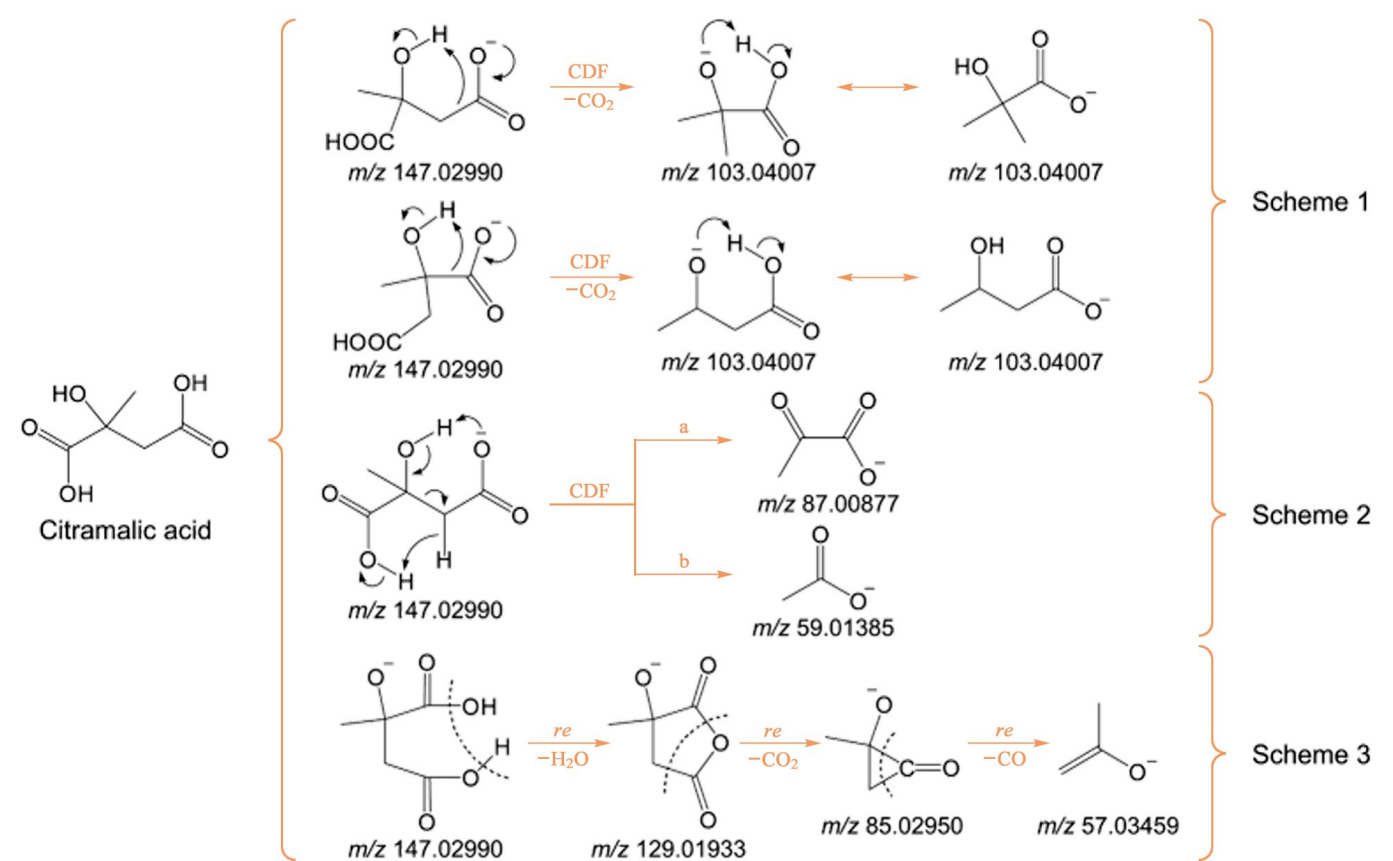
基于AI-Q-Orbitrap高碰撞解离的去质子化柠檬酸的3种裂解途径^[22]^

以上裂解规律的推理和特征质荷比片段的分析主要是通过手动管理非目标数据,即删除重复特征和化学噪声后整理成一个简明的特征列表,这是一项耗时且容易出错的工作。DeFelice等^[23]^设计了一个Web应用程序MS-FLO,工作流程如图2所示,该程序可作为一种自动程序来识别峰值表中错误数据,以提高数据初始处理后特征列表的正确率,同时利用保留时间校准、精确的质量公差、Pearson相关分析和峰高相似性来识别离子加合物、重复峰报告和同位素特征峰。结果表明,MS-FLO自动质谱特征优化程序可以提高后续统计数据的准确度以及非靶标筛查的效率。

**图2:**
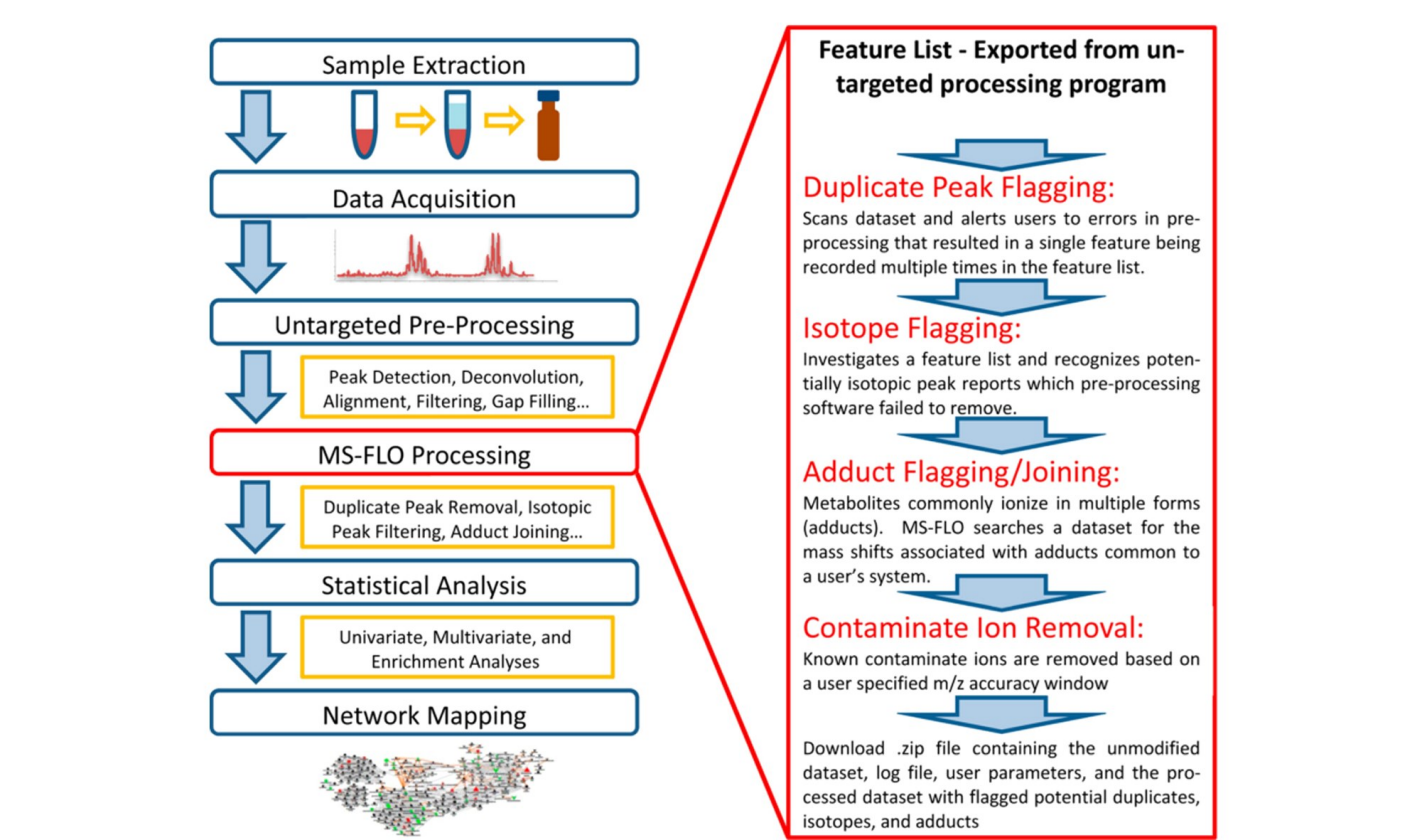
质谱特征列表优化器用于生成和处理非靶向LC-MS代谢组学数据库的工作流程^[23]^

### 2.2 质谱预测

由于现有质谱数据库和筛查模型的限制,在没有参考标准品的情况下,对未知分子结构的解析仍具有挑战性。机器学习是对大量数据进行分析和学习,从而让计算机系统自主挖掘数据之间的联系和相似性,并进行决策和预测的过程。这种高级算法技术可以用来监管食品安全质量,同时为食品质量等级的标准制定做出重大贡献。Gredell等^[24]^将机器学习与快速蒸发电离质谱法的数据相结合,比较了8种不同的机器学习算法对每个模型集的预测精准度,包括:(1)具有线性核、径向核和多项式核的支持向量机,(2)随机森林,(3)最近邻算法,(4)线性判别分析,(5)惩罚判别分析,(6)极端梯度提升,(7)逻辑提升,(8)偏最小二乘判别分析。同时生成了预测模型,并以质量等级、生产背景、品种类型和肌肉嫩度的标准对牛肉质量进行分类。结果表明,根据预测精准度评估的最佳机器学习算法因不同的牛肉分类标准而有差异,这表明直接用快速蒸发电离——“一刀切”的方法来判断肉类质量是不严谨的。由此可见,机器学习与MS数据相结合建立的预测模型补充了食品质量分类分级标准。

机器学习是通过已有的MS数据对未来的趋势变化进行推断。而质谱树是从MS数据中获取裂解顺序和关系,通过计算生成质谱树来预测未知分子产生碎片的路径。质谱树可以将分子裂解路径与(子)结构关系按层次顺序连接起来,通过对结构和子结构进行解析,识别化合物的分子式、碎片离子的元素组成和中性丢失,并预测分子指纹图谱,质谱树预测方法如图3所示。Goldman等^[25]^开发了一种质谱树识别代谢物的策略,并展示了预测的结果如何更好地识别检索任务中的未知成分。为了从不同的化合物中找到相似的裂解路径,需要对质谱树进行比对,具有相似裂解模式的化合物具有较强的化学相似性。结果表明,质谱树预测方法有利于未知代谢物鉴定和检索数据库搭建。

**图3:**
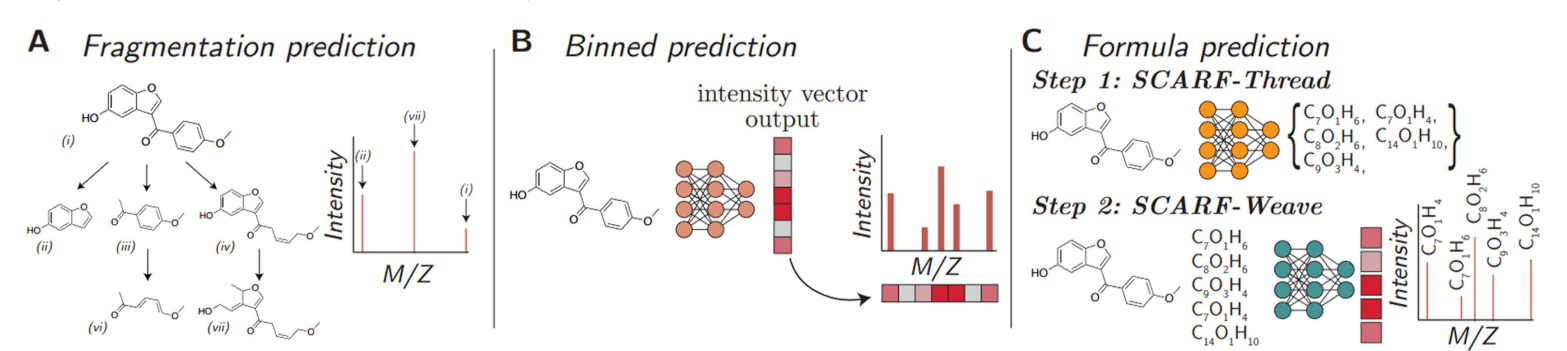
质谱树的3种预测方法示意图^[25]^

## 3 微型质谱仪

微型质谱仪在保留完整质谱功能的同时,去除了繁琐的样品前处理过程,具有更低的功耗特性,也更具有价格优势。为了适应现场监测的便携性,分析仪器的小型化与AMS分离技术的结合已经成为许多科学领域的关注点,让大多数样品在现场电离,更适用于非专业操作者使用。与实验室大型质谱仪要求的高真空系统相比,微型质谱仪既可以耦合非敞开式电离源,也可以耦合敞开式电离源^[26]^。因此真空系统和大气压接口的设计成为各种类型微型质谱仪研制的关键。

Gerbig等^[27]^使用微型质谱仪联合3种AMS(电喷雾电离法、解吸电喷雾电离法和低温等离子体电离法)测定了3种牛奶、5种鱼类和2种咖啡的化学指纹图谱。研究使用多元统计方法鉴定食品概况,评估了不同的数据处理方法获得的指纹图谱在食品认证中的适用性。结果表明,微型质谱仪-AMS和统计分析相结合的方法适用于食品的现场实时分析。Blokland等^[28]^评估了一些最新的AMS技术检测食品污染物(如农药、兽药和天然毒素)的潜力,目的是在保持质谱最佳灵敏度和稳健性的同时最小化检测所需的样品量。研究将基质辅助电离、手持式解吸常压化学电离、实时传输模式直接分析和涂层叶片喷雾分别耦合到台式单四极杆、三重四极杆和Q-Orbitrap质谱分析仪上。最终基于实验数据准确性的考虑,微型离子阱或三重四极杆质谱将成为未来食品安全快检领域便携式质谱串联的最低要求。

## 4 总结与展望

随着多种MS新技术的发展,数据库以及机器预测范围的大幅增加,未来研制出具备高分辨分离分析性能、集在线质谱耦合技术与高分辨筛查技术于一身的微型质谱仪十分可能。虽然微型质谱仪已在食品安全、消费品安全、公共安全等多个领域取得了很大进展,但仍然存在许多挑战,包括:(a)非均相样品的采样与分析;(b)复杂样品成分导致的离子抑制影响定量准确性;(c)大气压气流对质谱检测器造成的真空冲击;(d)检测受到温度、湿度、样品接触面积等因素影响较大。以上干扰均可能导致食品风险控制中假阳性结果的出现。因此,研发新型高选择性表面功能化改性材料,定向偶联到厘米级电离芯片上,实现微型质谱仪富集-分离-电离的一体化,可有效消除基质干扰,提高原位检测的准确性。同时,研制具有稳定梯度压力分布的小型多级真空系统以及低气压下的高效离子传输与聚焦技术,对实现快速、稳定、高灵敏、高分辨率的小型原位装置十分必要。

## 作者团队简介

张峰首席专家团队设在中国检验检疫科学研究院和国家市场监管重点实验室(食品质量与安全)。自2016年以来,团队得到了迅速发展。在首席专家的带领下,研究组聚焦食品中未知有害物的质谱侦测技术开发、食品实时质谱检测关键元件与技术开发、食品组学风险判定技术开发及食品安全质谱成像技术开发,承担了多项国家级科研项目,并与国内外相关学术机构建立了良好的合作关系。

课题组网站: https://www.caiq.org.cn/spaqyjs/index.shtml

**Figure:**
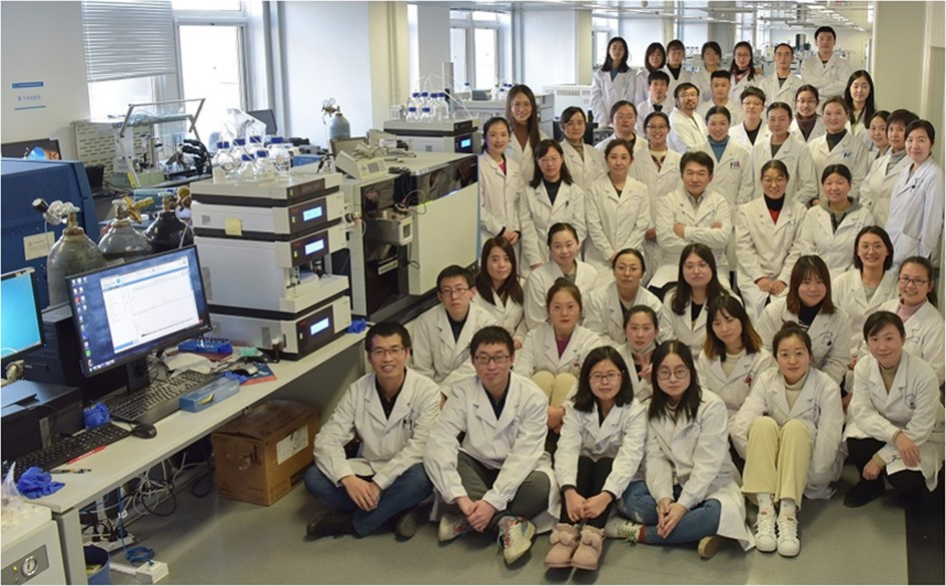


### 人才队伍

**首席专家:** 张峰研究员,中国检验检疫科学研究院副院长;国务院食品安全委员会专家委员会委员,中国检验检测学会副会长;国家“高层次人才特殊支持计划”科技创新领军人才

**团队成员:** 研究员9人,副研究员5人,助理研究员及研究实习员14人,在站博士后2人,在读博士、硕士研究生22人,共计52人

**团队精神:** 敢于理论创新,勇于技术突破,保障国家利益

### 科研项目及成果

**科研项目:** “十三五”以来,主持欧盟“地平线2020”项目、国家重点研发计划项目、国家自然科学基金项目等25项

**科研成果:** 制定国家标准及行业标准30余项,获得发明专利授权30余件,近5年在*Anal Chem*, *J Agric Food Chem*等期刊发表文章200余篇,主编著作10余部,获得国家二级标准物质2个,获得软件著作权7项

**获奖情况:** 以第一完成人获得“国家科技进步奖”二等奖、国家质检总局“科技兴检奖”一等奖、“中国分析测试协会科学技术奖(CAIA奖)”一等奖、“全国创新争先奖”(获奖人:张峰)等30余项奖励

### 研究领域

**Figure:**
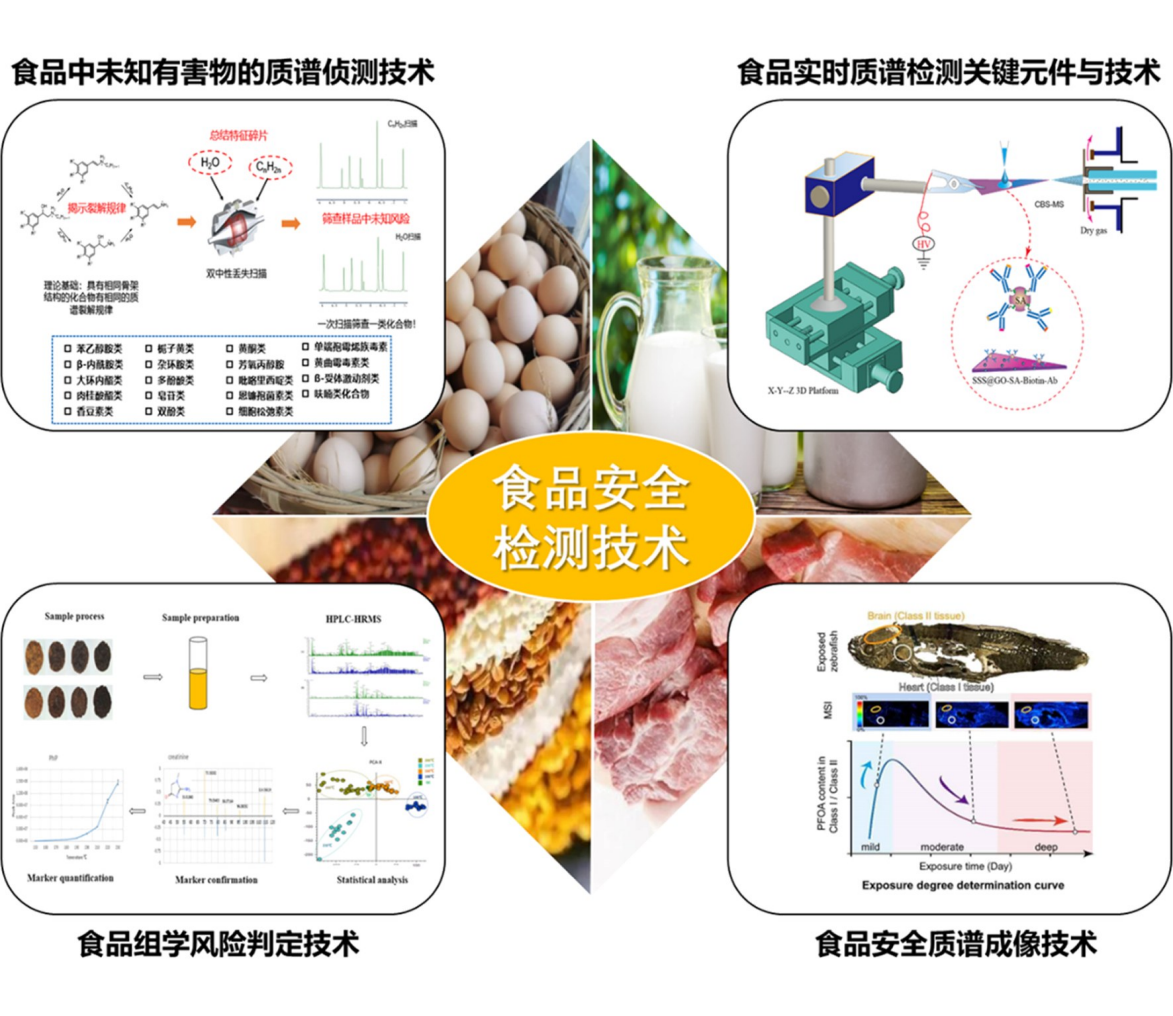


### 仪器设备

**Figure:**
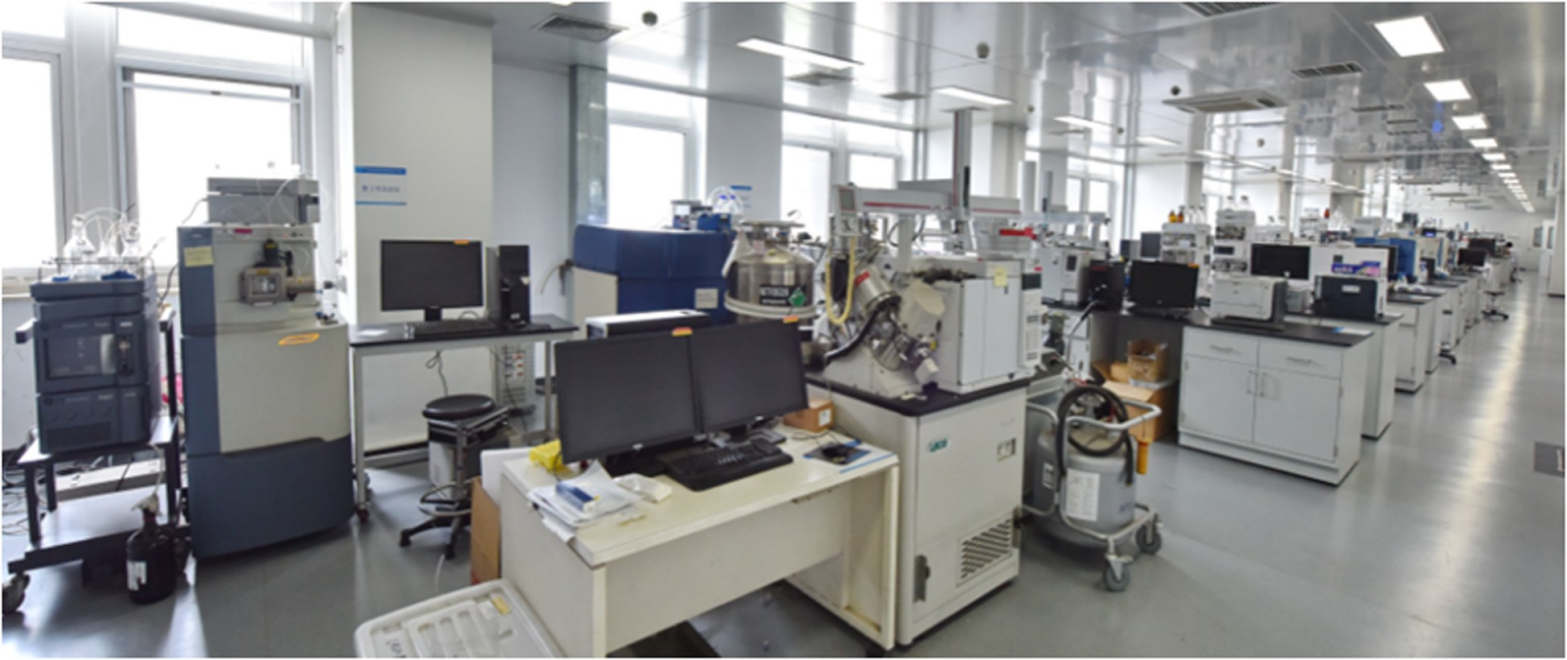


实验室面积3000 m^2^,拥有质谱显微镜、三合一高分辨质谱仪、环形离子淌度色谱-质谱联用仪、同位素质谱仪、高分辨静电场轨道阱质谱仪、高分辨飞行时间质谱仪、三重四极杆质谱仪、电感耦合等离子体质谱仪、气相色谱仪、液相色谱仪、凝胶色谱仪、离子色谱仪等设备270余台(套),原值超2亿元人民币
